# 17α Hydroxylase/17,20 lyase deficiency: clinical features and genetic insights from a large Turkey cohort

**DOI:** 10.1007/s12020-024-03962-6

**Published:** 2024-07-17

**Authors:** Zeynep Siklar, Emine Camtosun, Semih Bolu, Melek Yildiz, Aysehan Akinci, Firdevs Bas, İsmail Dündar, Asli Bestas, Edip Ünal, Pinar Kocaay, Tulay Guran, Gonul Buyukyilmaz, Aylin Kilinc Ugurlu, Buşra Gurpinar Tosun, Ihsan Turan, Erdal Kurnaz, Bilgin Yuksel, Doga Turkkahraman, Atilla Cayir, Gamze Celmeli, E. Nazli Gonc, Beray Selver Eklioğlu, Semra Cetinkaya, Seniha Kiremitci Yilmaz, Mehmet Emre Atabek, Muammer Buyukinan, Emrullah Arslan, Eda Mengen, Esra Deniz Papatya Cakir, Murat Karaoglan, Nihal Hatipoglu, Zerrin Orbak, Ahmet Ucar, Nesibe Akyurek, Emine Demet Akbas, Emregül Isik, Sare Betul Kaygusuz, Zumrut Kocabey Sutcu, Gulcan Seymen, Merih Berberoglu

**Affiliations:** 1 Ankara University Faculty of Medicine, Department of Pediatric Endocrinology, Ankara, Türkiye; 2 Inonu University Faculty of Medicine, Department of Pediatric Endocrinology and Diabetes, Malatya, Türkiye; 3 Bolu Abant Izzet Baysal University Faculty of Medicine, Department of Pediatric Endocrinology, Bolu, Türkiye; 4 Istanbul University Faculty of Medicine, Department of Pediatric Endocrinology, Istanbul, Türkiye; 5 Dicle University Faculty of Medicine, Department of Pediatric Endocrinology, Diyarbakır, Türkiye; 6 Ankara Bilkent City Hospital, Clinic of Pediatric Endocrinology, Ankara, Türkiye; 7 Marmara University Faculty of Medicine, Department of Pediatric Endocrinology and Diabetes, Istanbul, Türkiye; 8 Cukurova University Faculty of Medicine, Department of Pediatric Endocrinology, Adana, Türkiye; 9 Ankara Etlik City Hospital, Clinic of Pediatric Endocrinology, Ankara, Türkiye; 10 University of Health Sciences, Antalya Training and Resarch Hospital, Clinic of Pediatric Endocrinology, Antalya, Türkiye; 11 University of Health Sciences, Erzurum Training and Resarch Hospital, Clinic of Pediatric Endocrinology and Diabetes, Erzurum, Turkey; 12 Hacettepe University Faculty of Medicine, İhsan Doğramacı Children’s Hospital, Department of Pediatric Endocrinology, Ankara, Türkiye; 13 Necmettin Erbakan University Faculty of Medicine, Department of Pediatric Endocrinology, Konya, Türkiye; 14 University of Health Sciences, Dr Sami Ulus Child Health and Diseases Training and Research Hospital, Clinic of Pediatric Endocrinology, Ankara, Türkiye; 15 University of Health Sciences, Haseki Training and Research Hospital, Clinic of Pediatric Endocrinology, Istanbul, Türkiye; 16 Selcuk University Faculty of Medicine, Department of Pediatric Endocrinology, Konya, Türkiye; 17 Ege University Faculty of Medicine, Department of Pediatric Endocrinology and Diabetes, Izmir, Türkiye; 18 Ankara Training and Research Hospital, Ankara, Türkiye; 19 University of Health Sciences, Bakirkoy Dr. Sadi Konuk Training and Resarch Hospital, Clinic of Pediatric Endocrinology, Istanbul, Türkiye; 20 Gaziantep University Faculty of Medicine,, Department of Pediatric Endocrinology, Gaziantep, Türkiye; 21 Erciyes University Faculty of Medicine, Department of Pediatric Endocrinology, Kayseri, Türkiye; 22 Ataturk University Faculty of Medicine, Department of Pediatric Endocrinology and Diabetes, Erzurum, Türkiye; 23 University of Health Sciences, Şişli Hamidiye Etfal Training and Resarch Hospital, Clinic of Pediatric Endocrinology, İstanbul, Türkiye; 24 Baskent University Faculty of Medicine, Department of Pediatric Endocrinology, Konya, Türkiye; 25 Bursa Dortcelik Children’s Hospital, Bursa, Türkiye; 26 Gaziantep Children’s Hospital, Gaziantep, Türkiye; 27 Kahramanmaras Necip Fazil City Hospital, Kahramanmaraş, Türkiye; 28 Basaksehir Cam and Sakura City Hospital, Clinic of Pediatric Endocrinology, İstanbul, Türkiye; 29 University of Health Sciences, Umraniye Training and Research Hospital, Clinic of Pediatric Endocrinology, İstanbul, Türkiye

**Keywords:** 17α hydroxylase, 17,20, lyase deficiency, *CYP17A1*, delayed puberty, congenital adrenal hyperplasia, final height

## Abstract

**Purpose:**

17α Hydroxylase/17,20 lyase deficiency (17OHD) is a rare form of congenital adrenal hyperplasia, typically diagnosed in late adolescence with symptoms of pubertal delay and hypertension. This study aimed to determine the clinical and laboratory characteristics of 17OHD cases and gather data on disease management.

**Methods:**

Data from 97 nationwide cases were analyzed using the CEDD-NET web system. Diagnostic, follow-up findings, and final heights of patients were evaluated.

**Results:**

Mean age at admission was 13.54 ± 4.71 years, with delayed puberty as the most common complaint. Hypertension was detected in 65% at presentation; hypokalemia was present in 34%. Genetic analysis revealed Exon 1–6 homozygous deletion as the most frequent mutation, identified in 42 cases. Hydrocortisone replacement was universal; pubertal replacement was administered to 66 cases. Antihypertensive treatment was required in 57 (90%) patients. Thirty-seven cases reached final height, with an average SD of 0.015 in 46,XX and −1.43 in 46,XY. Thelarche and pubarche did not develop properly in some cases despite estradiol treatment.

**Conclusion:**

This study represents the largest cohort of pediatric cases of 17-hydroxylase deficiency (17OHD) documented in the literature. Hypertension and hypokalemia can serve as guiding indicators for early diagnosis.The final height is typically considered to be normal. The relationship between genotype and phenotype remains elusive. The initial genetic test for exon 1–6 deletions may be MLPA in our region.

## Introduction

17α Hydroxylase/17–20 lyase deficiency (17OHD) is a rare autosomal recessive form of congenital adrenal hyperplasia (CAH) caused by biallelic mutations in the CYP17A1 gene, encoding cytochrome p450c17. It accounts for about 1% of all CAH forms, with an occurrence rate of 1 in 50,000 newborns [1]. P450c17 has 17α-hydroxylase and 17,20-lyase activities, essential for glucocorticoid and sex steroid biosynthesis [2]. Mutations in CYP17A1 lead to decreased cortisol and sex steroid production, resulting in sexual infantilism and pubertal failure, with increased mineralocorticoid precursors causing hypertension and hypokalemia [2–4]. Insufficient dehydroepiandrosterone sulfate (DHEA-S) production impairs adrenarche and development of pubic and axillary hair [3]. Despite normal anti-Müllerian hormone (AMH) production in 46,XY patients, steroid synthesis is impaired [5].

17OHD can present as complete or partial forms, with most cases being complete, diagnosed at later ages due to mineralocorticoid excess alongside androgen and estrogen deficiency [1, 6]. The CYP17A1 gene, comprising eight exons, translates into a 508 amino acid polypeptide, with over 150 different mutations identified. The prevalence of 17OHD is higher in Brazil, China, and Japan, often due to the founder effect, though genotype-phenotype correlation remains unclear [6–10].

This multicenter study aims to evaluate all 17OHD cases based on clinical, laboratory, and long-term follow-up findings and to gather data on patient management, addressing the lack of comprehensive national-level data [11–21].

## Materials and methods

This nationwide study was conducted using a web-based system. A case recording form (CRF) was used to collect the clinical and laboratory findings of the patients. The CRF was designed by two physicians (Z.Ş, M.B.) and was uploaded to the CEDD-NET Web Registry System website (https://cedd.saglik-network.org), and the consistency of all data entered into the system was checked. All pediatric endocrinology clinics in our country were asked to enter data of their patients on a voluntary basis. Participants from centers that were members of the Pediatric Endocrinology and Diabetes Association at the national level.

Data was recorded over a 12-month period (1 October 2021–31 June 2023). Of all 62 centers with pediatric endocrinologists, 24 of them reported that (in personal comminucation) they have no patient diagnosed with 17OHD. Thirty two centers entered data of 97 patients into the web system.

The data, collected retrospectively, consisted of physical examination, auxological findings, hormone assays, biochemical and genetic findings, additional features at follow-up, treatment, and final height data. From this data, family histories*, presenting complaints* at the time of diagnosis and clinical and laboratory findings of the patients were evaluated.

Height and weight were measured, body mass index (BMI) was calculated using the standard formula [weight in kg/(height in m)], and the respective standard deviation score (SDS) was calculated based on Turkish reference data [22]. Obesity was defined as BMI percentile for age z-score 1.64, equivalent to 95 th percentile [23].

Short stature is defined as a condition in which the height of an individual is two standard deviations (SD) below the corresponding mean height of a given age, sex and population group [24].

Delayed puberty is defined in girls by the lack of breast development at the age of 13 years or by the absence of menarche at the age of 15 years and in boys by the lack of testicular development above 3 mL at the age of 14.0 years [25]. Hypertension was defined if systolic BP and/or diastolic BP measurements were ≥95th percentile for age, height, and gender [26]. Hypokalemia was defined as a serum potassium level < 3.5 mEq/l. Bone age was evaluated using the Greulich-Pyle method according to karyotype [27]. Cortisol, oestradiol and testosterone levels were analysed by immunochemiluminometric assays (ICMA), while aldosterone and 17-hydroxyprogesterone levels were determined by radioimmunoassay (RIA), ACTH levels were quantified by fluoroimmunoassay (FIA), and sodium and potassium levels were assessed by routine biochemical assays.

The diagnosis of 17-OHD was established based on typical laboratory findings of “low cortisol and aldosterone, suppressed PRA, increased gonadotropins and progesterone, as well as absence of sexual steroid production” are findings supporting 17OHD. Detection of mutation in *CYP17A1* analysis confirms the diagnosis. The initial evaluation was conducted via next-generation sequencing, while MLPA analysis was employed in instances where the aforementioned method yielded negative results.

Treatment and follow-up characteristics were requested to be recorded which included treatments received, psychosocial evaluation, reared gender, presence of gonadectomy in 46,XY cases which were raised as female. In addition, information about the patient’s clinic and laboratory findings at the last follow-up and final height were evaluated.

Criteria for inclusion of patients in the study group:

1. Cases admitted to pediatric endocrinology clinic and diagnosed with 17OHD based on clinical and laboratory findings
2. All cases with genetically detected *CYP17A1* mutations

Exclusion criteria for patients in the study group:

1. Diagnosed with congenital adrenal hyperplasia other than 17OHD.

All data analyzes were performed with SPSS 16.0. Descriptive statistics were used to evaluate demographic and clinical characteristics. Data were defined as percent, mean ± standard deviation (SD), and median (IQR) —distance between the 25th and 75th percentiles—. “The Mann–Whitney U” test was employed to compare the medians of two independent groups, while “Student’s t-test” was utilized to compare the means of two independent groups. The Chi-Square test was used for comparing categorical variables. Statistically, p < 0.05 was considered significant.

The study adhered to the Helsinki Declaration of 1975. Ethical approval for the study was received from the Ankara University Human Research Ethics Committee (approval number: İ2-131-21).

## Results

### Admission characteristics

A total of 97 case data from 78 families were recorded in the study. Fiftynine (60,8%) of the cases have 46,XY karyotype and 38 (39,1%) have 46,XX karyotype. Among the cases with 46,XY karyotype, 6 of them presented with ambiguous genitalia. The average age of admission was found to be 13.54 years old, 12.71 years for those 46, XX, and 11.49 years for those 46, XY. Thirteen cases (13.4%) were between 0–5 years old, 12 cases (12.3%) were 5–10 years old, 43 cases (44.3%) were 10–15 years old, 29 cases (29.8%) were 15–18 years old. Of the 25 cases diagnosed at a young age under ten years of age, 13 were diagnosed as a result of screening due to a positive family history, and 6 were diagnosed while being examined for the presence of ambiguous genitalia.

At the time of admission, 94 of 97 cases were raised as female. The most common complaints of the cases were primary amenorrhea (n:46) and delayed puberty (n: 45) (Table 1).

**Table 1:** The complaints and physical examination of the patients stated at first admission to the pediatric endocrinology clinic

Complaints	n (%)
• Primary amenorrhea	46 (47.4%)
• Pubertal delay	45 (46.3%)
• Ambiguous genitalia	6 (6.1%)
• Positive family history (for screening)	19 (19.5%)
• Others:	
∘ Obesity	5 (5.1%)
∘ Chronic hypokalemia	4 (4.1%)
∘ Short stature	2 (2%)
∘ Inguinal mass	3 (3%)
∘ Sustained hypertension	3 (3%)
∘ Adrenal insufficiency with hypoglycemic convulsion	3 (3%)
∘ Coincidentally detection of absence of Müllerian structure in abdominal US	1 (1%)
*Physical examination*	
• Hypertension	63 (%65)
• Hyperpigmentation	5 (%5)
Total Cases	97 (100%)

There was consanguinity between the parents in 87 patients within 78 family. Most of the patients had complete form (87/97), while 10 cases (10.6%) (4 of them 46,XX, 6 of them 46,XY) had partial form. Electrolytes and hormonal profile were similar in both. A 13,6 years old case with a 46,XY karyotype and female genital phenotype had breast development. In this case, gonadotropins were found to be high, but testosterone and estrogen levels were found to be low.

When the height SDS values of the cases at admission are calculated taking their karyotypes into account; in 46,XX cases, the mean height SD was −1,08 ± 1,54 SD, and in 46,XY cases, the mean SD was −0,58 ± 1,54 SD (Table 2). > When analyzing cases with height SDS > 2 SD and < −2 SD, it was observed that only one case with a 46,XX karyotype had a height SDS above 2 SD, whereas six cases fell below −2 SD. In contrast, none of the 46,XY cases had a height SDS above 2 SD, and 11 of these cases exhibited a height SDS below −2 SD.

**Table 2:** Antropometric measurements of patients on admission

	46,XX cases (n:38)	46,XY cases (n: 59)	All cases (n: 97)
Age of admission (year)	12.71 ± 3.61	11.49 ± 5.29	13.54 ± 4.71
Height SDS	−1.08 ± 1.52	−0.58 ± 1.56	−0.79 ± 1.55
BMI (kg/m^2^)	18.89 ± 3.18	18.74 ± 3.42	18.77 ± 3.31
BMI SDS	−0.58 ± 1.55	−0.36 ± 1.07	−0.47 ± 1.28

Values represent as Mean ± SD

Hyperpigmentation were reported only in 5 cases (5%). At presentation, hypertension was detected in 63 cases, accounting for 65% of the cohort. Among those with hypertension, 57 cases (90% of hypertension cases) received regular antihypertensive treatment. One patient with severe hypertension died shortly after diagnosis due to hypertensive encephalopathy. The duration of antihypertensive treatment was between 2 and 144 months (median 42.27 months).Three cases presented with severe hypoglycemia were monitored under a diagnosis of adrenal insufficiency before 17OHD diagnosis. Hyponatremia was not detected in any of them.

### Hormonal and biochemical evaluation

In laboratory data, basal cortisol was found to be low in all, and ACTH was high in 55 of 97 cases. Peak cortisol responses were inadequate (<18 mcg/dl) in patients (n:75) underwent ACTH stimulation test. Basal serum progesteron levels were high; serum 17-hydroxyprogesterone (17OH-P) varied widely from low to slightly high, while serum DHEAS levels were low in all patients. Testosterone or estradiol levels were also blunted. Elevated gonadotropins LH and FSH are typically detected during the pubertal period (Table 3).

**Table 3:** Laboratory data of the cases on admission

	Median (IQR)
Basal cortisol (mcg/dl)	1.80(5–54)
Stimulated Cortisol (mcg/dl)	5.9(6–8.3)
ACTH (pg/ml)	34(127–910)
Progesterone (ng/ml)	1.37(6.9–8.25)
DHEAS (µg/mL)	150(150–622)
Na (mEq/L)	140(139–141)
K (mEq/L)	3.8(3.2–4.4)
LH (mIU/ml)	2(3.3–4)
FSH (mIU/ml)	3.7(8.7–7.7)
PRA (mcg/L/hr)	1.4(0.3–5.4)

Values represent as Mean ± SD and Median(IQR)

Hypokalemia (< 3.5 mmol/L) was detected in 33 cases (34%). While the age at diagnosis was 13.1 ± 3.8 years in cases with hypertension; it was 12.3 ± 3.9 years in those without hypertension (p:0.07). The mean age at diagnosis was also similar between cases with and without hypokalemia (12.8 ± 4.1 years vs. 12.2 ± 4.1 years, p:0.08).

### Mutation characteristics

**Fig. 1:**
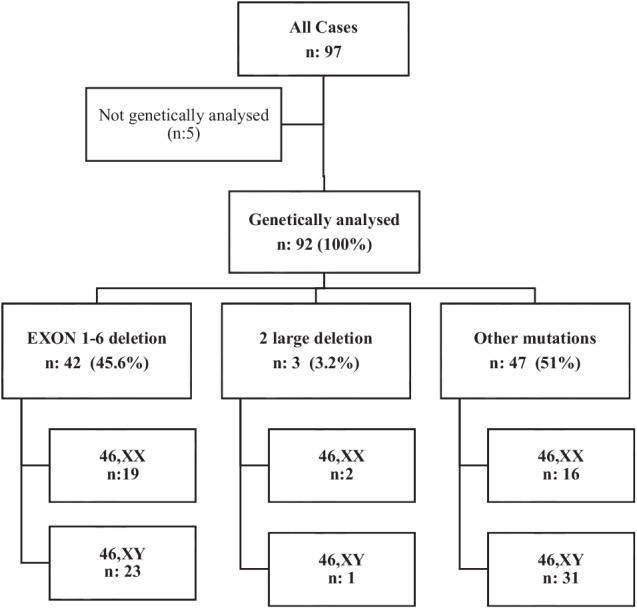
Distribution of cases with genetic evaluation according to mutation group

**Fig. 2:**
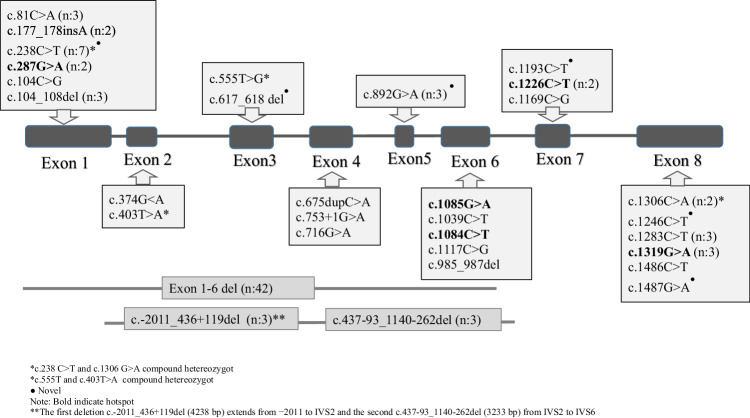
Mutations detected in 92 cases where genetic analysis was performed

A homozygous mutation was detected in the *CYP17A1* gene for all screened cases (n:92). The most common mutation was Exon 1–6 homozygous deletion (n: 42, 45.8%). Additionally, 2 different large deletions (The first deletion c.-2011_436+119del (4238 bp) extends from −2011 to IVS2 and the second c.437-93_1140-262del (3233 bp) from IVS2 to IVS6) were found in 3 siblings which were previously reported.^17^ In other cases, different mutations, mainly point mutations (missense, frameshift, etc.), were identified (Fig. 1). Mutations other than large deletions are most frequently concentrated in Exon 1 and Exon 6 (Fig. 2).

Patients with large deletions and other mutations were compared. No difference was detected in terms of age at admission, anthropometric characteristics, and presence of hypertension (Table 4).

**Table 4:** Comparison of cases with and without large deletions

	Large deletions (n: 45)	Other mutations (n: 47)	p value
Age of diagnosis (year)	12.5 ± 4.1	11.95 ± 5.4	0.36
Height SDS	−0.67 ± 1.7	−1.01 ± 1.32	0.29
BMI (kg/m2)	19 ± 2.8	19.07 ± 5	0.55
Hypertension (%)	55%	53.5%	0.98*
Basal Cortisol (mcg/dl)	0.59 (0.4–1)	0.82 (0.47–1.85)	0.09**
Stim. Cortisol (mcg/dl)	0.68 (0.5–1)	1.8 (0.7–3.7)	0.001**
ACTH (pg/ml)	154 (105–382)	201 (108–439)	0.44**
Progesterone (ng/ml)	8.5 (6–10)	7.2 (4.9–10.7)	0.67**
Na (mEq/L)	140.2 ± 2.6	140.1 ± 2.7	0.84
K (mEq/L)	3.6 ± 0.75	3.75 ± 0.53	0.45
PRA (mcg/L/hr)	0.56 (0.20–1.57)	0.70 (0.20–2.24)	0.10**

Values represent as Mean ± SD and Median(IQR)

* Chi-Square test statistic

** Mann–Whitney U statistic

*p* value <0.05 is shown in italics and bold

### Treatment and follow-up characteristics

The cases were followed between 0.1 and 16.2 years (mean 4.52 years). Hydrocortisone replacement was started in all cases at diagnosis. Almost all patients reached the normal biochemical profile after cortisone replacement. Pubertal replacement therapy was started in 66 cases (68%). During the follow-up period, pubarche did not develop in 28 of these cases, thelarche remained at stage 2 in 5 cases, and at stage 3 in 10 cases despite proper estradiol replacement. Considering the cases that had used replacement treatments for at least two years and reached their final height; in 9 cases telarche remained in Stage 2 and Stage 3, in 25 cases it reached Stage 4 and Stage 5. Again, while pubarche remained in stage 1 in 10 of the cases that reached final height, it remained in stage 2 (n: 8) and stage 3 (n: 7) in 15 cases. Pubarche could be reached stage 4 and stage 5 in 12 cases.

Gonadectomy was performed in 33 of the 46,XY cases. Two of these were performed during operations for inguinal hernia before 17OHD diagnosis. Leydig cell tumor was detected in one case. The tumor was described as well-circumscribed, 2.5 × 1.5 × 1.2 cm in size, within the testicular tissue.

When the reared gender was evaluated, only 2 of the patients (with 46,XY karyotype) were being raised as males. A gender decision had not yet been made for another 46,XY infant with ambiguous genitalia.

Psychiatric evaluation was performed in 26 cases. Depression was detected in 4 cases, while the cases who underwent psychiatric evaluation did not have sexual identity problem who were raised as females.

### Final height data

Thirty-seven (38.5%) cases reached their final height. Sixteen of these cases had 46,XX and 21 had 46,XY karyotype. Final height SDS was normal in both 46,XX and 46,XY patients. There were no patients whose final height was taller than 2 SDS.

While there was no difference between the height SDS of the patients at the time of admission according to karyotype; final height SDS was higher in the 46,XX karyotype patient than in the 46,XY patient. Additionally, when calculated as ∆HSDS, the height gain was much greater in 46,XX patients than in 46,XY patients (Table 5).

**Table 5:** Characteristics of cases (n:37) who attained to final height

	46,XX cases (n: 16)	46,XY cases (n: 21)	p values
Final height (cm)	163.2 ± 5.54	167.35 ± 6.59	–
Height SDS on admission	−1.3 ± 1.62	−0.86 ± 1.94	0.25
On admission number of cases with Height SDS < −2SD and > 2 SD (n)	Height SDS < −2SD →(3) Height SDS > 2 SD→(1)	Height SDS < −2SD →(7) Height SDS > 2 SD→(0)	–
Final height SDS	0.015 ± 0.94	−1.43 ± 1.06	***<0.01***
Number of cases with Final Height SDS < −2SD and > 2 SD (n)	Height SDS < −2SD →(0) Height SDS > 2 SD→(0)	Height SDS < −2SD →(7) Height SDS > 2SD→(0)	–
∆HSDS	1.3 ± 1.62	−0.86 ± 1.94	***0.0008***
Final BMI (kg/m2)	23.09 ± 4.81	24.05 ± 4.36	0.26
Final BMI SDS	0.15 ± 1.71	0.042 ± 1.38	0.41

Values represent as Mean ± SD

*p* value <0.05 is shown in italics and bold

## Discussion

Our study constitutes the largest cohort reported in the literature to date and consists of cases admitted in the pediatric age group. To date, there are very few articles containing case series on 17OHD, other than individual case reports. Case series with a number of cases between 5 and 10 have been reported, especially from Asia [28–32]. These few case series reports generally included descriptive clinical and genetic features of the patients. Publications with more than 10 cases were made from China, Brazil and Turkey. Most of the reported cases involve adult cases [2, 6–9, 11, 19, 33–35].

We observed that the cases were mainly admitted after the age of 10 with complaints of delayed puberty. Typically, in 17OHD, insufficient androgen and estrogen production and delayed onset and insufficient progression of pubertal signs are observed. Hypertension is uncommon in infancy due to low renal mineralocorticoid sensitivity. On the other hand, does not show signs of glucocorticoid deficiency, although it is a lack of cortisol synthesis and diagnosis shifts to the adolescence period. Hence, the most common presentation of 17OHD is an adolescent girl without secondary sexual characteristics or menses and low-renin hypertension [3, 15].

Most of the cases diagnosed in the younger age group were investigated as a result of family screening or because ambiguous genitalia. In most of our cases, there were consanguinity between the parents. The possibility that this autosomal recessive problem occurs more frequently in consanguineous marriages should not be ignored.

In cases with 17OHD, hyperpigmentation may be observed due to increase in ACTH []. However, in most of our cases hyperpigmentation was not reported. hyperpigmentation could be mild and therefore not noticed in most of the cases during admission. Although the rarity of the disease could be a reason why the diagnosis was not considered, the fact that hyperpigmentation was not noticeable in a significant portion of these patients could play a role in diagnostic delays.

The most common reason for admission in our cases (who grew up as females) was delayed puberty. This could be explained by the fact that 46,XY cases were diagnosed at a similar age to 46,XX cases, which were compatible with the female phenotype with the reason for presentation was delayed pubertal characteristics.

Among our cases, partial 17OHD was detected at a low rate. Most of the 17OHD cases reported in the literature were complete forms [6]. Cases with 46,XY karyotype with ambiguous genitalia and 46,XX karyotype with insufficient onset and progression of puberty could be stated as having partial 17OHD clinic [1]. Again, in partial forms, hypertension and hypokalemia may not be observed [11]. Also it has been reported that the hormonal profile is similar in complete and partial forms, such as our cases. But could be more severe in complete forms [1].

Isolated 17,20 lyase deficiency was not detected in any case of our cohort. This is significantly rare in the literatüre [36].

Considering the height SDS calculated according to the karyotypes; most of the patients had normal height. It was reported that, in 17OHD cases, short stature was not an expected finding, as sex steroid deficiency delays the closure of the epiphyses. Reported cases were often being normal or tall. However, if the height evaluations of these cases were calculated disregarding the karyotype, but according to the female gender they were raised with, it could be interpreted differently. In addition, we concluded that 17OHD cases usually have normal height at admission and short stature could be observed.

When laboratory data were evaluated, our 3 patients presented with hypoglycemia were considered to have adrenal insufficiency as their initial diagnosis. Although there is a decrease in cortisol biosynthesis in 17OHD, it is compensated physiologically because of the corticosterone excess stimulated by increased ACTH. As a glucocorticoid, increased corticosterone replaces cortisol and leads to the absence of clinical adrenal insufficiency [1].

Although serum sodium levels were normal, hypokalemia was seen as one of the prominent features of the disease and even caused the patients to be followed up with the diagnosis of isolated hypokalemia. In a hypokalemic case, the presence of accompanying hypertension should be a warning for the diagnosis of 17OHD.

At diagnosis, nearly 2/3 of our cases had hypertension. In 17OHD, the increase in mineralocorticoid precursors clinically causes the development of hypertension and hypokalemia. A small number of 17OHD cases (10–15%) are normotensive. While the rate of hypertension was 88% in a series of 16 cases published in Brazil, the rate was reported as high as 95.6% in a publication from China and including the adult age group (11, 33, 2). The main cause of hypertension is high levels of DOC. However, corticosterone and its 5 alpha metabolites, 11-oxygenated progesterone derivatives are factors that contribute to the development of sodium retention and hypertension [30, 37].

From a physiological perspective, it is anticipated that the administration of hydrocortisone will result in the effective management of hypertension. Nevertheless, approximately half of the hypertensive cases continued to require antihypertensive treatment, comprising calcium channel blockers and/or spironolactone.

It is suggested that in cases where hypertension persists, other factors may be effective in the occurrence and maintenance of hypertension. Since high DOC levels generally saturate mineralocorticoid receptors in 17OHD cases, the severity of clinical features and the age of onset of hypertension may differ even among patients with the same mutation [3].

The diagnosis of 17OHD cases typically occurs during puberty, and the duration of uncontrolled hypertension can be prolonged. This condition carries the risk of causing end-organ damage [2]. Therefore, early diagnosis and effective management of hypertension are critically important. In the study of Zhao, they reported that blood pressure could not be controlled in 13,8% of the patients with hypertension, and a significant portion of the patients did not use regular antihypertensive treatments [2]. Severe hypokalemia may also contribute to damaging renal tubular epithelial cells. The degree of hypokalemia also depends on the degree of mineralocorticoid production and indicates the severity of 17OHD.

In this study, we found no significant difference in the ages of individuals with hypertension or hypokalemia compared to those without these conditions. This finding challenges the existing literature, which has reported the opposite trend. According to the research, these two main symptoms can help in the early identification and diagnosis of patients [19].

More than 150 mutations have been detected in the *CYP17A1* gene to date. Although certain mutations are more prevalent in some countries, the phenotype-genotype relationship has not yet been clearly established. It has been stated that even in the presence of the same mutation, there may be significant differences in the severity of the disorder [6–9, 38]. Approximately half of our cases, of which genetic analysis was performed, had exon 1–6 deletions. The finding suggested that it would be appropriate to request MLPA analysis as a first step in our country.

In cases with large deletions, it can be expected that the degree of enzyme deficiency can be severe and gonadal steroid production can be more affected. However, we could not detect any difference in terms of reference laboratory characteristics between these two mutation groups.

All cases reared female needed estrogen replacement at pubertal age. Despite appropriate estrogen replacement therapy, thelarche remained insufficient in some of the cases. As it is known, 17OHD cases are exposed to the effects of high progesterone for a long time before diagnosis, and this have negative effects on endometrium and breast tissue. Thus, in some patients, breast tissue may not respond to estrogen therapy [39].

In 17,20-lyase deficiency, since the adrenal results in deficiency of DHEA and DHEAS, it is an expected finding that the adrenarche is inadequate [3]. In all our cases, pubarche did not occur on admission. However, after estrogen replacement, pubarche was observed in some cases. The onset and progression of pubarche seemed to be compatible with the view that estrogen might influence hair follicles in these cases [21].

Gonadal tumors have rarely been reported in 17OHD cases. As a gonadal pathology, a Leydig cell tumor was detected in one case. Leydig cell tumor is a rare non-germ cell tumor which accounts for 1–3% of testicular neoplasms. Its etiology is not clearly known [30]. Both Leydig cell hyperplasia and Leydig cell tumor can be seen in testosterone biosynthesis disorders, and have also been reported in 17OHD cases [40–43]. In those cases, gonadotropins stimulation of Leydig cells may lead to Leydig cell hyperplasia and the development of Leydig cell tumors in later stages.

It was an interesting finding that in one case with 46,XY (female phenotype) karyotype had spontaneous breast development despite having low levels of testosterone and estrogen. It has been reported that a patient with complete 17OHD had breast tissue growth, gonadal steroids were low, and gonadotropins were high. Spontaneous breast development in the case could be due to detectable estrogen levels, and it was also stated that Leydig cell hyperplasia was detected in this case [38]. Although the spontaneous breast development seen in our case could be related to possible Leydig cell hyperplasia, it could also be considered as the equivalent of gynecomastia seen in cases with hypergonadotropic hypogonadism.

Psychiatric evaluation was performed in a small number of cases. We think that the clinician should be more aware of this issue and more psychiatric evaluations of the cases should be made.

During follow-up, 37 cases reached final height. To date, final height data are available in case report articles and very few cases. Additionally, karyotype is often not taken into account in final height SDS calculations. Our study includes the highest number of cases reported so far that reached final height. Another strength is that final height data is given according to karyotype. In all 46,XX cases, the final height SD values were within normal values and an improvement of height SDS was approximately 1.5 SD. It was stated that in some of these cases, the growth rate increased with sex steroid replacement, and they reached normal height [11]. But similar height improvement was not observed in 46,XY cases despite mean final height SDS were normal. Also, none of the cases reached final height was tall.

The study had a limitation, including the inability to obtain homogeneous results for all data. Despite this limitation, the study’s strengths are notable. It successfully reached many centers across the country, making it the largest cohort in the literature for this condition. The study provided comprehensive insights into the genotype-phenotype relationship within our country. Additionally, final height data could be evaluated for a significant proportion of cases.

As a result, the number of cases with 17OHD in our country was considerable and seemed to be related to the high number of consanguineous marriages. Pubertal delay was the dominant phenotype. Hypertension and presence of hypokalemia were important diagnostic clues. The frequency of Exon 1–6 deletion was almost half of the cases in our country. According to their karyotypes, final height of 17OHD cases were usually normal.
